# Biodegradable Chitosan-Based Ambroxol Hydrochloride Microspheres: Effect of Cross-Linking Agents

**DOI:** 10.4103/0975-1483.76414

**Published:** 2011

**Authors:** HH Gangurde, NV Chavan, AS Mundada, DV Derle, S Tamizharasi

**Affiliations:** 1 *SNJB’s Shriman Suresh Dada Jain College of Pharmacy, Neminagar, Chandwad, Nasik, Maharastra, India*; 2 *Department of Pharmaceutical Sciences, N.D.M.V.P. Samaj’s, College of Pharmacy, Gangapur Road, Shivaji Nagar, Nasik - 422 002, Maharastra, India*; 3 *NANDHA College of Pharmacy, Department of Pharmaceutics, Perundurai Road, Rode, Tamil Nadu, India*

**Keywords:** Ambroxol hydrochloride, chitosan microspheres, cross-linking agents

## Abstract

The objective of this study was to investigate the influence of type of cross-linking method used on the properties of ambroxol hydrochloride microspheres such as encapsulation efficiency, particle size, and drug release. Microspheres were prepared by solvent evaporation technique using chitosan as a matrix-forming agent and cross-linked using formaldehyde and heat treatment. Morphological and physicochemical properties of microspheres were then investigated by scanning electron microscopy (SEM), X-ray diffractometry (XRD), differential scanning calorimetry (DSC), and Fourier-transform infrared spectroscopy (FTIR) spectroscopy. The cross-linking of chitosan takes place at the free amino group because of formation of imine bond as evidenced by FTIR. The DSC, XRD, and FTIR analysis showed that chitosan microspheres cross linked by heating were superior in properties and performance as compared to the microspheres cross-linked using formaldehyde. SEM results revealed that heat-treated microspheres were spherical, discrete having smooth, and porous structure. The particle size and encapsulation efficiencies of the prepared chitosan microspheres ranged between 10.83–24.11 μm and 39.73μ80.56%, respectively. The drug release was extended up to 12 h, and the kinetics of the drug release was obeying Higuchi kinetic proving diffusion-controlled drug release.

## INTRODUCTION

Ambroxol is an active *N*-desmethyl metabolite of the mucolytic bromohexine. It is indicated for acute and chronic disorders of respiratory tract, where there is copious thick secretion or mucus production. It has biological half-life of 3–4 h. It is absorbed throughout gastrointestinal tract (GIT) showing oral bioavailability of about 70–72%. Usual initial dose of ambroxol hydrochloride is 30 mg thrice a day. Therefore, to reduce frequency of dosing as well as to increase bioavailability and enable better patient compliance, formulating sustained release dosage form was necessary.[1–4] Several sustained release formulations of ambroxol hydrochloride have been reported.[5–7]

Chitosan is a biodegradable *N*-deacetylated product of the polysaccharide, Chitin. It has been used by a number of authors as a matrix-forming material in microspheres because of its biodegradable nature, nontoxicity, biocompatibility, and mucoadhesivity.[8–11] These multiparticulate solid dosage forms have a number of advantages such as more uniform distribution of the drug in the GIT, more uniform drug absorption, reduced local irritation, and elimination of unwanted intestinal retention of polymeric material. Different methods such as ionotropic gelation,[8,12] spray drying,[13,14] emulsification-solvent evaporation[15,16] are mentioned in the literature for preparation of chitosan microspheres.

In this study, we prepared chitosan microspheres using solvent evaporation techniques and evaluated the effect of methods of cross-linking such as cross-linking by chemical such as formaldehyde[17] and cross-linking by heat treatment[18] onto the encapsulation efficiency, particle size, and drug release. The cross-linked microspheres from the optimized formulations were then characterized by FTIR, DSC, XRD, and SEM.

## MATERIALS AND METHODS

### Materials

Ambroxol hydrochloride was obtained as a gift sample from Glenmark Pharmaceutical Ltd., Nasik and chitosan from Central Fisheries Research Institute, Kochi, India. Acetic acid and formaldehyde (35% w/v) solutions were purchased from S.D. Fine Chemicals, Mumbai, India. All other chemicals used were of analytical grade.

### Methods

#### Preparation of chitosan microspheres

Initially, chitosan (100 mg) was dissolved in 2 %v/v acetic acid solution. The drug (100 mg) was then added to it, and the suspension was extruded through syringe in 200 mL of 1:1 mixture of heavy and light liquid paraffin containing 1 %w/v SPAN80. The resulting emulsion was then stirred using propeller stirrer (Remi’s Equipment, Mumbai) at 500 rpm. After 30 min, the developed microspheres were cross-linked chemically (formaldehyde—FA) or by the heat treatment (H), as explained follows:

- To produce the FA-crosslinked chitosan microspheres, 1 mL of formaldehyde was added to emulsion and stirring was continued for 6 h. Cross-linked microspheres thus formed were separated by filtration, washed repeatedly with hexane, followed by water to remove the paraffin, acid, and the excess of the cross-linking agent. Microspheres were prepared with varying ratios of chitosan: drug like 1:1, 2:1, 3:1, and 4:1 and designated as, FA-1, FA-2, FA-3, and FA-4, respectively.
- To produce the heat treatment cross-linked chitosan microspheres, the temperature of the emulsion was raised up to 60°C and stirred at 500 rpm overnight. The microspheres were then separated by filtration and washed repeatedly with hexane followed by water to remove acid and paraffin. The resulting microspheres prepared with varying ratios of chitosan: drug like 1:1, 2:1, 3:1, and 4:1 were designated as H-1, H-2 and H-3, and H-4, respectively.

#### Percentage yield of microspheres

Percent yield value calculated as per the total raw material taken as a theoretical amount and the total weight of microspheres taken as practical value

$$\mathrm{Percent}\;\mathrm{yield}\;\mathrm{value}\;=\;\frac{Practical\;value}{Theoretical\;value}\times 100$$

#### Drug entrapment efficiency

Weighed quantity of microspheres were crushed and suspended in distilled water for 24 h to extract the drug from microspheres. The filtrate was then analyzed at 244.4 nm using UV–Vis spectrophotometer (JASCO V630, Japan) for drug content. The encapsulation efficiency was calculated using following equation:

$$\mathrm{Encapsulation}\;\mathrm{efficiency}\;=\;\left(\mathrm{Drug}\;\mathrm{entrapped}/\mathrm{Theoretical}\;\mathrm{drug}\;\mathrm{content}\right)\;\times \;100$$

#### Particle size analysis of microspheres

Average particle diameter and size distribution of microspheres were determined by laser diffractometry using a Mastersizer Micro V 2.19 (Malvern Instruments, Malvern, UK). Approximately, 10 mg of microspheres were dispersed in 10 mL distilled water containing 0.1 %v/v Tween 80 for 1 min using an ultrasonic bath. Then, an aliquot of the microspheres suspension was added into recirculation unit, which was subsequently circulated 3500 times/min. Particle size was expressed as equivalent volume diameter. The analysis was carried out in triplicate. The particle size distribution was also expressed in terms of SPAN factor determined as:

$$\mathrm{SPAN}\;=\;\frac{d_{90}\;-\;d_{50}}{d_{10}}$$

where *d*_10_, *d*_50_, and *d*_90_ are the diameter sizes, and the given percentage value is the percentage of particles smaller than that size. A high SPAN value indicates a wide size distribution.[14]

#### Scanning electron micrography

The microspheres were scanned using scanning electron microscope (Leica-Stereoscan-440). For the SEM, the microspheres were mounted directly on to the SEM sample slab using double-sided sticking tape and coated with gold film thickness of 200 mm under reduced pressure of 0.001 mmHg. The shape and surface characteristic of the microspheres was observed under electron microanalyzer and photographs were taken using SM 4504 camera.

### X-ray diffractometry

ray powder diffractometry was carried out to investigate the effect of microencapsulation process on crystallinility of drug. Powder XRD pattern was recorded on XRD (Philips-PW-1050) with filter Ni, Cu Kα radiation, voltage 40 kV, and a current of 20 mA. The scanning rate employed was 1°C/min over the 5° to 50° diffraction angle (2θ) range. The XRD patterns of drug powder, polymer, and drug-loaded microspheres were recorded.

#### Differential scanning calorimetry

The DSC analysis of pure drug, polymer, and drug-loaded microspheres was carried using DSC (Mettler TC 11, TA Processor). The samples (6 mg each) were placed into a pierced aluminum sample container. The studies were carried out under a static air atmosphere in the temperature range of 50–500°C, at a heating rate of 10°C/min. The peak temperatures were determined after calibration with standard.

### Fourier-transform infrared spectroscopy

Drug–polymer interactions were studied by FTIR spectroscopy. The spectra were recorded for pure drug, polymer, and drug-loaded microspheres using FTIR spectrophotometer (Jasco FTIR-410). Samples were prepared by KBr disk method and the scanned over the range of 400–4000 cm^-1^, and the resolution was 2/cm.

### *In vitro* drug release studies

Microspheres equivalent to 75 mg ambroxol hydrochloride were filled in a capsule[13] and *in vitro* drug release was studied using USP Apparatus I with 900 mL of dissolution medium at 37.5 ± 0.1°C for 12 h at 100 rpm. Then, 0.1 N HCl (pH 1.2) was used as dissolution medium for the first 2 h, followed by pH 6.8 phosphate buffer for further 10 h. Five milliliters mL of sample was withdrawn after every hour and was replaced with an equal volume of fresh dissolution medium. Collected samples were analyzed at 244.4 nm spectrophotometrically. The study was performed in triplicate. Dissolution study was also conducted for marketed capsule Mucolite® SR (M1).

### Release kinetics

Data obtained from *in vitro* release studies were fitted to various kinetics equations to find out the mechanism of drug release from microspheres. The kinetics models used were zero order, first order, and Higuchi models. The rate constants were also calculated for the respective models.

## RESULTS AND DISCUSSION

This study was undertaken to evaluate the effect of the method of cross-linking onto the performance of chitosan microspheres. Chitosan was cross-linked chemically using formaldehyde or thermally by heat treatment. Ambroxol hydrochloride was chosen as the model drug.

### Morphology and drug encapsulation efficiency

**Figure 1:**
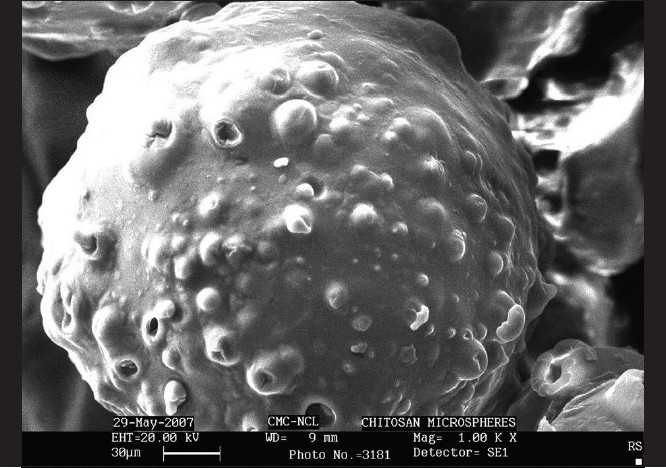
Scanning electron micrograph of optimized H3 microsphere at 1.00× magnification

Thermally cross-linked (H3) microspheres were found to be spherical, discrete, and smooth surfaced with distinct pores as compared to the microspheres cross-linked using formaldehyde Figure 1. Drug encapsulation efficiency was found to be 39.73–58.65% for thermally cross-linked microspheres whereas it was 68.45–80.56% for chemically cross-linked ones Table 1. The high-drug encapsulation for formaldehyde cross-linked microspheres could be because of formation of more strong and rigid matrix as it involves actual chemical reaction between formaldehyde and chitosan.

**Table 1:** Physical characteristics of the ambroxol hydrochloride loaded microspheres

Formulation code	Drug: polymer ratio	% Yield	Entrapment efficiency	Mean particle size (µm)	SPAN
FA1	1:1	85.98	68.45 ± 0.745	10.83 ± 0.035	2.146
FA2	1:2	91.45	76.20 ± 0.234	13.81 ± 0.124	2.854
FA3	1:3	89.26	78.57 ± 1.024	16.95 ± 0.078	1.934
FA4	1:4	79.44	80.56 ± 0.928	17.91 ± 0.382	2.301
H1	1:1	95.34	39.73 ± 0.640	18.02 ± 0.463	2.289
H2	1:2	92.68	40.98 ± 1.245	20.82 ± 1.082	2.147
H3	1:3	88.25	45.23 ± 0.735	22.27 ± 0.846	2.032
H4	1:4	80.56	58.65 ± 0.862	24.11 ± 0.628	1.778

### Effect on particle size

Particle size for thermally cross-linked chitosan microspheres was found to be 18.00–24.11 µm and for chitosan microspheres cross-linked by formaldehyde was found to be 10.83–17.91 µm. It was seen that the particle size of microspheres increased as the concentration of polymer increased, and this may be because of the fact that increase in polymer concentration causes increase in the viscosity of inner phase, which leads to increased cross-linking. This increased matrix density of polymer may result in the increase in the particle size of the microspheres. As cross-linking by formaldehyde was more rigid and strong, particle size of such microspheres was small as compared to thermally cross-linked microspheres. SPAN factor ranged in between 1.77 and 2.289, indicating narrow size of distribution.

### *In vitro* drug release

**Figure 2:**
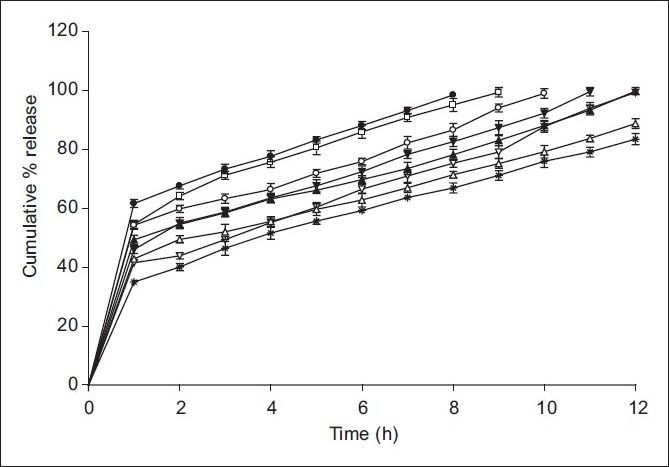
*In vitro* dissolution profile of ambroxol hydrochloride-loaded chitosan microspheres. ● H1; ○ H2; ▲ H3; ∆ H4; □ F1; ▼ F2, F3; * F4

*In vitro* dissolution study revealed that microspheres showed sustained drug release beyond 8 h. It was clear from Figure 2 that drug-release rate decreased as the chitosan concentration increased. The drug release from the developed microspheres was biphasic showing initial burst effect (rapid drug release) followed by slow diffusion of drug. It was also seen that drug release from these biodegradable microspheres was also dependent upon the type and rigidity of matrix. It was already stated that cross-linking by formaldehyde forms more rigid and strong matrix due to covalent imine bond formation between aldehyde group of formaldehyde and the amino groups of chitosan.[12] Therefore, the drug release through FA cross-linked microspheres was slow as compared to thermally cross-linked microspheres.

*In vitro* dissolution results showed that the H3 and FA3 microspheres maintained better-sustained action up to a period of 12 h. However, cross-linking by chemical agent like formaldehyde is harmful because the traces of this toxic chemical in the chitosan microspheres can cause irritation to mucosal membranes and also it is difficult to ensure that the chitosan microspheres are absolutely free from the cross-linking agent. Hence, H3 microspheres were considered as optimized formulation and it was used further evaluated for compatibility study between drug and the polymer and surface characterization.

### Kinetic treatment of dissolution data

All the formulations followed Higuchi’s kinetics with R2 ranging from 0.991 to 0.999 Table 2. The mechanism of drug release was analyzed by Korsmeyer-Peppas equation using n value. The value of *n* varies between 0.22 and 0.35, which showed Fickian diffusion. The value of kinetic constant show decreased trend from 4.87 to 3.83 with increased polymer concentration.

**Table 2:** Release kinetic of ambroxol hydrochloride-loaded chitosan microspheres

Formulation code	Kinetic models							
	Zero-order		First-order		Higuchi model		Peppas model	
	R^2^	K_0_ (%mg/h)	R^2^	K_0_ (h-1)	R^2^	K^h^ (%mg/h1/2)	R^2^	n
FA1	0.9851	4.981	0.927	0.3525	0.9979	33.136	0.9907	0.2710
FA2	0.9765	4.873	0.8115	0.2745	0.9955	30.042	0.9605	0.3123
FA3	0.9691	4.790	0.7689	0.2685	0.9969	28.676	0.9524	0.3896
FA4	0.9909	4.045	0.9777	0.1186	0.9946	24.110	0.9783	0.3595
H1	0.9859	4.611	0.8238	0.4016	0.9991	34.835	0.9603	0.2248
H2	0.9502	4.483	0.8398	0.2832	0.9915	31.328	0.9122	0.26
H3	0.9563	4.210	0.8902	0.1789	0.9934	28.827	0.926	0.2798
H4	0.9696	3.830	0.9341	0.1335	0.997	25.637	0.9426	0.2901

**Figure 3:**
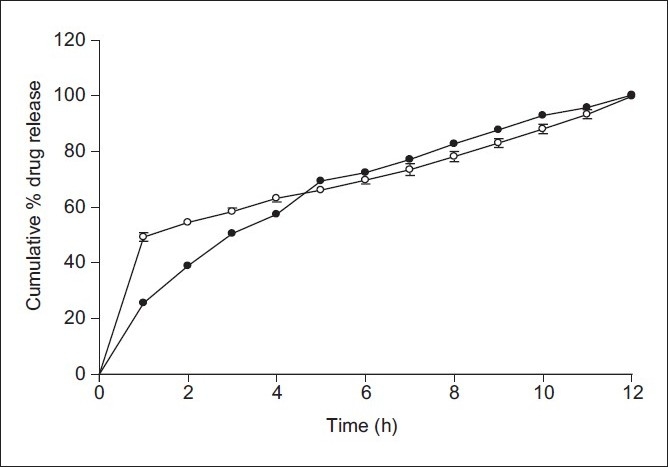
Comparative *in vitro* dissolution profile of optimized H3 microspheres with marketed capsule M1. ● M1; ○ H3

Similarity factor (*f2*) and difference factor (*f1*) were calculated for optimized microspheres considering marketed capsule as the reference standard. It was found that *f1* and *f2* value for H3 were 51.83 and 3.06, respectively. This suggested that H3 microspheres showed similarities of dissolution profiles with that of marketed capsule Figure 3. T90% of H3 and marketed capsule was 10.3 and 9.48 h, respectively, which suggested that these microspheres showed release profiles comparable with that of marketed capsule.

### X-ray diffractometry

**Figure 4:**
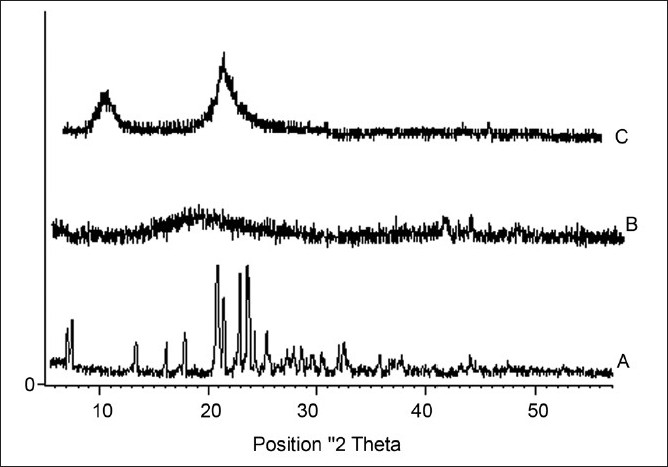
X-ray diffractograms of ambroxol hydrochloride (A), chitosan (B), chitosan-loaded ambroxol hydrochloride loaded microspheres (C)

Characteristic crystalline peaks of ambroxol hydrochloride was absent in the XRD of ambroxol hydrochloride-loaded chitosan microspheres which suggested that drug might be in amorphous nature in microspheres Figure 4.

### Differential scanning calorimetry

**Figure 5:**
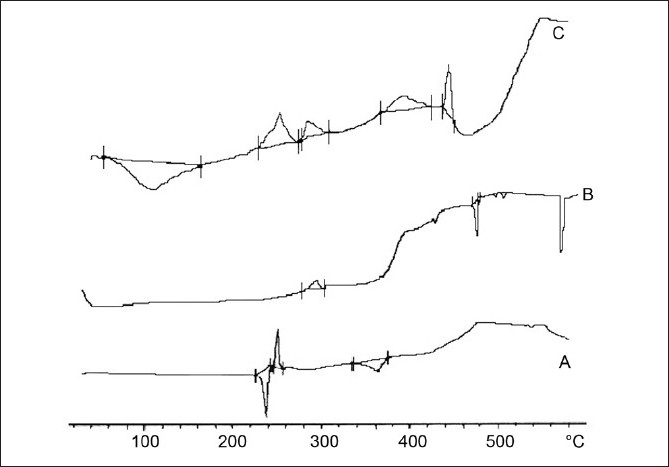
DSC thermograph of ambroxol hydrochloride (A), chitosan (B), chitosan-loaded ambroxol hydrochloride loaded microspheres (C)

Endothermic peak of pure ambroxol hydrochloride at 243°C was absent in ambroxol-loaded chitosan microsphere, which suggested that drug might have shown molecular dispersity in microspheres Figure 5.

### Fourier transform infra-red spectroscopy

Characteristic peak of chitosan polymer were located at 3430 cm^-1^ for OH group, 1654 cm^-1^ for carbonyl stretching vibration, and at 1616 cm^-1^ for amino group. In case of drug-loaded chitosan microspheres peak at 1719 cm^-1^ might be due imine bond formed in cross-linked chitosanFigure 6. However, the peaks of drug not obtained exactly in drug-loaded microspheres, which suggested that nature of drug might be changed to amorphous.

**Figure 6:**
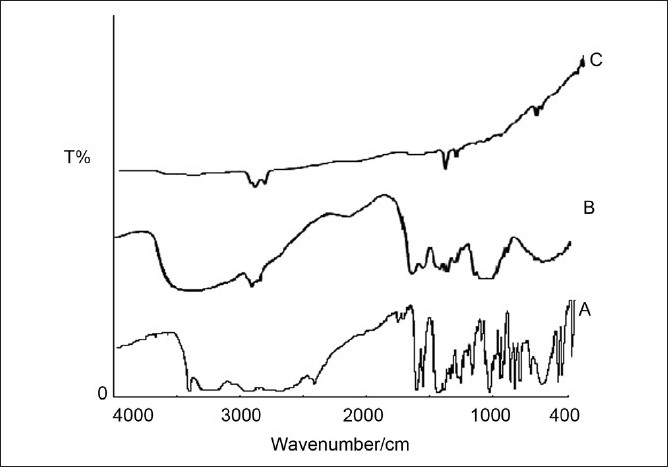
FTIR spectra of ambroxol hydrochloride (A), chitosan (B), chitosan-loaded ambroxol hydrochloride-loaded microspheres (C)

## CONCLUSION

This study was designed to evaluate the effect of the method of cross-linking onto the performance of chitosan microspheres and from the outcome of the investigation it can be concluded that though formaldehyde gives better cross-linking effect for chitosan microspheres, however, due to its toxic nature and inability to remove its traces from the microspheres, one has to think about the alternative method for cross-linking and heat treatment is found to be better alternative as it could control the drug release to the desired extent and resulted in excellent microspheres with narrow size distribution.
